# A third dose of inactivated SARS-CoV-2 vaccine induces robust antibody responses in people with inadequate response to two-dose vaccination

**DOI:** 10.1093/nsr/nwac066

**Published:** 2022-04-14

**Authors:** Taicheng Zhou, Tianpei Shi, Ao Li, Lingzhi Zhu, Xinshuai Zhao, Naiyin Mao, Wanting Qin, Hanfang Bi, Mei Yang, Muxian Dai, Fengwei Liu, Rong Wang, Wei Su, Liang Zhang, Wenbo Xu, Jia Wei, Zijie Zhang

**Affiliations:** Central Laboratory and Liver Disease Research Center, The Affiliated Hospital of Yunnan University, Yunnan University, China; State Key Laboratory for Conservation and Utilization of Bio-resource and School of Life Sciences, Yunnan University, China; State Key Laboratory of Genetic Resources and Evolution, and Yunnan Laboratory of Molecular Biology of Domestic Animals, Kunming Institute of Zoology, Chinese Academy of Sciences, China; College of Life Science, University of Chinese Academy of Sciences, China; State Key Laboratory for Conservation and Utilization of Bio-resource and School of Life Sciences, Yunnan University, China; State Key Laboratory for Conservation and Utilization of Bio-resource and School of Life Sciences, Yunnan University, China; State Key Laboratory for Conservation and Utilization of Bio-resource and School of Life Sciences, Yunnan University, China; NHC Key Laboratory of Medical Virology and Viral Diseases, National Institute for Viral Disease Control and Prevention, Chinese Center for Disease Control and Prevention, China; State Key Laboratory for Conservation and Utilization of Bio-resource and School of Life Sciences, Yunnan University, China; State Key Laboratory of Genetic Resources and Evolution, and Yunnan Laboratory of Molecular Biology of Domestic Animals, Kunming Institute of Zoology, Chinese Academy of Sciences, China; College of Life Science, University of Chinese Academy of Sciences, China; State Key Laboratory for Conservation and Utilization of Bio-resource and School of Life Sciences, Yunnan University, China; State Key Laboratory for Conservation and Utilization of Bio-resource and School of Life Sciences, Yunnan University, China; Central Laboratory and Liver Disease Research Center, The Affiliated Hospital of Yunnan University, Yunnan University, China; Central Laboratory and Liver Disease Research Center, The Affiliated Hospital of Yunnan University, Yunnan University, China; State Key Laboratory for Conservation and Utilization of Bio-resource and School of Life Sciences, Yunnan University, China; State Key Laboratory of Genetic Resources and Evolution, and Yunnan Laboratory of Molecular Biology of Domestic Animals, Kunming Institute of Zoology, Chinese Academy of Sciences, China; College of Life Science, University of Chinese Academy of Sciences, China; State Key Laboratory for Conservation and Utilization of Bio-resource and School of Life Sciences, Yunnan University, China; Central Laboratory and Liver Disease Research Center, The Affiliated Hospital of Yunnan University, Yunnan University, China; NHC Key Laboratory of Medical Virology and Viral Diseases, National Institute for Viral Disease Control and Prevention, Chinese Center for Disease Control and Prevention, China; Central Laboratory and Liver Disease Research Center, The Affiliated Hospital of Yunnan University, Yunnan University, China; State Key Laboratory for Conservation and Utilization of Bio-resource and School of Life Sciences, Yunnan University, China

Mass application of SARS-CoV-2 vaccines is a major approach to reduce SARS-CoV-2 transmission and COVID-19 symptoms worldwide. The inactivated SARS-CoV-2 vaccine is one of the most widely administered vaccine types in many countries, with over 2 billion doses administered as of 31 August 2021. However, clinical trials estimate 65%–85% protection from detectable symptoms for inactivated SARS-CoV-2 vaccines [1–4], with 15%–35% of people remaining insufficiently protected after two-dose immunization. Immune protection for these ‘non-responders’ is critical for population immunity as established by mass vaccination. A third dose of inactivated (CoronaVac) vaccine has been shown to boost antibody response by between 3–5 folds in the general population [5]. Whether a third dose can induce an adequate antibody response in ‘non-responders’ remains unclear. Here we report a trial of a third dose in a cohort of 105 vaccinated participants (Supplementary Fig. S1) with suboptimal antibody responses (non-responders) after two doses (43.8% were men; average age 43 ± 11 years [± SD]; average BMI 23.5 ± 3.4 [± SD], Supplementary Table S1).

Our non-responder cohort were selected from an immunogenicity screening of over 2031 participants who had received two doses of inactivated SARS-CoV-2 vaccine (Supplementary Table S1). Using the competitive inhibition method to measure neutralization antibody levels, we observed a 95.1% seroconversion rate, with 4.9% of people lacking detectable neutralizing antibodies two weeks after the completion of two-dose vaccination (Fig. 1A). Khoury *et al*. analyzed *in vitro* neutralizing antibodies with clinical protection data in seven vaccine clinical trials and estimated 50% protection against detectable infection to be 20.2% of the mean convalescent neutralizing antibody level [6]. Thus, we used 20.2% mean convalescent neutralizing level as the cut-off for clinical protection in our analysis and found a 19.7% negative rate (Fig. 1A). The distribution of SARS-CoV-2-specific immunoglobulin-G (IgG) is similar to that of neutralizing antibodies, while immunoglobulin-M (IgM) responded to vaccination in a passive manner, with low seroconversion rate (Supplementary Fig. S2).

**Figure 1.**
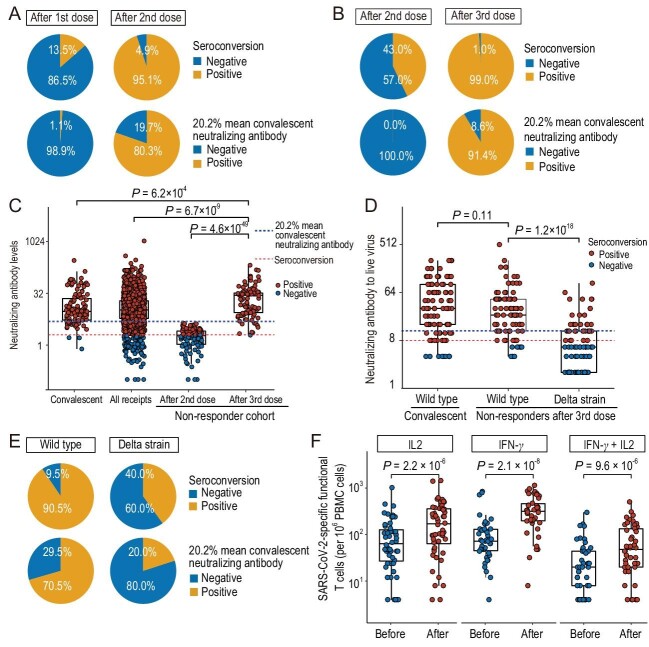
Immune response to a third dose of inactivated vaccine in non-responders. (A) The seroconversion rate of neutralizing antibodies after the first dose and after the second dose. (B) The seroconversion rate of neutralizing antibodies after the second dose and after the third dose in our third-dose-trial cohort. Neutralizing antibody levels were measured using the competitive inhibition method. (C) Neutralizing antibody levels of non-responders after the third and second dose, of the general population after the second dose, and of COVID-19 convalescent patients. Each point represents an individual volunteer. (D) Neutralizing antibody titers measured by live virus test for convalescent patients (wild-type virus) and non-responders after the third dose (wild-type and Delta variant). (E) The seroconversion rate of neutralizing antibodies after the third dose as measured by live wild-type and Delta-variant viruses. (F) The SARS-CoV-2-specific T cell responses (cells producing both IL-2 and IFN-γ in response to SARS-CoV-2 peptide pool) before and after the third dose in randomly selected third-dose recipients. The third dose was significantly correlated with an increase in T cell responses (*P* = 2.2 × 10^–6^ between IL-2 groups; *P* = 2.1 × 10^–8^ between IFN-γ groups; *P* = 9.6 × 10^–6^ between IFN-γ + IL-2 groups).

To test whether a third dose can induce an adequate antibody response in people with suboptimal immunogenicity, we recruited 105 qualified volunteers with a neutralizing antibody level below the cut-off for 50% clinical protection [6]. These participants included 54 who were seronegative after two doses and 51 who were seropositive but not reaching the 50% clinical protection cut-off. Participants received the third dose 4–25 weeks after the second dose. We found that a third dose of CoronaVac in non-responders had a substantially higher neutralizing level compared to the primary immunization (Supplementary Fig. S3). We found that the neutralizing antibody levels of 104 non-responders (99.0%) passed seropositive cut-off and 96 (91.4%) passed the estimated cut-off for 50% protection against detectable infection two weeks after receiving the third dose (Fig. 1B). Out of 54 previously seronegative volunteers, 53 converted to seropositive and 46 out of 51 seropositive but sub-optimally protected volunteers gained neutralizing antibody levels higher than the 50% protection threshold after the third dose. The geometic mean titer (GMT) of neutralizing antibodies was 19 after the third dose, a 12-fold increase from the second dose (*P* = 4.6 × 10^–49^, Supplementary Table S2). In comparison, a third dose in the general population induced a 4.9-fold increase in neutralizing antibody GMT [5]. The GMT of neutralizing antibodies in the non-responder cohort after the third dose was ∼1.8-fold higher than the GMT in the general population after the second

 

dose (*P* = 6.7 × 10^–9^) and 1.4-fold higher than the GMT in convalescent COVID-19 patients (*P* = 6.2 × 10^–4^, Fig. 1C, Supplementary Table S2). Analyzing virus-specific IgG and IgM revealed that the increase in neutralizing antibodies after the third dose was likely attributed to IgG, while virus-specific IgM did not change much from the second to the third dose (Supplementary Table S2).

We next applied a live virus neutralizing test to further validate the neutralization level in 95 third-dose recipients using wild-type SARS-CoV-2 and the Delta variant (Fig. 1D). The live virus test and the competitive inhibition method gave a highly correlated neutralization level (Pearson correlation coefficient = 0.84, Supplementary Fig. S4A). Consistently, the seropositive rates using seroconversion cut-off and 50% protection cut-off were 90.5% and 70.5%, respectively (Fig. 1E), confirming that most non-responders gained protection against wild-type SARS-CoV-2 after a third dose. However, the neutralizing assay using the live Delta variant revealed significantly decreased neutralization activity compared to the wild-type virus (*P* = 1.2 × 10^–18^, Fig. 1D), with 40% and 20% seropositive rate using seroconversion cut-off and 50% protection cut-off, respectively (Fig. 1E). Compared to the general population (GMT = 143.1 [95%CI 110.8–184.7]) [5], the third dose in non-responders elicited a significantly lower neutralizing antibody level (GMT = 25.9 [95%CI 21.1–31.8], Supplementary Table S3). These data suggest a third dose of inactivated SARS-CoV-2 vaccine is an effective strategy to gain humoral immune protection in people who respond inadequately to the two-dose vaccination protocol. However, the neutralizing antibodies elicited by a third dose is relatively low and offers limited protection against the Delta variant.

We then analyzed factors that may affect humoral immunity gain induced by a third dose of inactivated vaccine in the non-responder cohort. Similar to a third dose in the general population [5], the interval between the second and third dose was significantly correlated with the increase in neutralizing antibody level after the third dose (R^2^ = 0.07, *P* = 0.0053, Supplementary Fig. S5A). Conversely, age, BMI and underlying health conditions could not explain variations in the neutralizing antibody level increases (Supplementary Fig. S5).

To understand the T cell responses in non-responders, we performed FluoroSpot assays for 66 participants to measure T cell immunity against SARS-CoV-2. We found that most participants showed detectable SARS-CoV-2-specific polyfunctional T cells (interferon-γ [IFN-γ] and interleukin-2 [IL-2] secreting T cells upon SARS-CoV-2 peptide pool stimulation) before receiving the third dose despite insufficient antibody responses among these people (Fig. 1F). It has been reported that the CD4 T cell response is highly correlated with the severity of COVID-19 symptoms [7]. Thus, a prevalence of SARS-CoV-2-specific polyfunctional T cells in people with poor humoral immune response might explain the clinical trial observation that most inactive vaccine recipients are protected from severe symptoms although some recipients are not protected from infection [2–4]. After the third dose, there was an overall increase in SARS-CoV-2-specific polyfunctional T cell counts (52 vs. 24 median IFN-γ + IL-2 double-secreting cells per 10^6^ peripheral blood mononuclear cells (PBMCs), *P* = 9.6 × 10^–6^; 252 vs. 80 median IFN-γ-secreting cells, *P* = 2.1 × 10^–8^; 174 vs. 96 median IL-2-secreting cells, *P* 2.2 × 10^–6^, Fig. 1F), suggesting that a third dose can promote Th1 cell responses.

To further dissect the T cell responses in CD4+ and CD8+ cells, we performed activation-induced marker (AIM) assays [8]. Consistent with FluoroSpot results, 87.5% and 45% of people exhibited detectable CD4 and CD8 T cell responses to SARS-CoV-2 peptide pools, respectively. This confirms that most non-responders have acquired CD4 T cell immunity against SARS-CoV-2 despite poor antibody responses. However, unlike FluoroSpot results that mainly represent Th1-type T cells, SARS-CoV-2-specific T cells in overall CD4 and CD8 T cells remain largely unchanged before and after the third dose (Supplementary Fig. S6A). Interestingly, we found that some samples showed similar levels of CD4 and CD8 T cell response, while a significant proportion of individuals tended to exhibit predominately CD4 T cell responses (Supplementary Fig. S6B). These data revealed a heterogeneity in the orchestration of CD4 and CD8 responses to the inactivated vaccine in non-responders.

In the safety assessment, we found that systemic muscle pain and headaches tended to be less common after the third dose compared to the first two doses (Supplementary Fig. S7A). Conversely, local (injection-site) adverse reactions were generally more common after the third dose than the first two doses (Supplementary Fig. S7B). No grade three or four events were reported in our third-dose cohort.

Many countries are launching mass application of the third dose as a ‘booster’ dose, but whether a third dose elicits an adequate antibody response among non-responders is unknown. Our results confirmed the value of a third dose among non-responders, as most non-responders gained neutralizing antibodies, reaching seropositive status. However, the antibody level elicited by a third dose in this special population is not adequate for neutralizing the Delta variant. The weakened protection against this variant of concern in non-responders is likely due to their defective antibody response compared to good-responders [5]. Thus, understanding the molecular basis underlying the impeded response to an inactivated SARS-CoV-2 vaccine is critical for improving protection among non-responders.

We observe that neutralization against the Delta variant is linearly correlated with the neutralizing antibody titer of the wild-type virus (Pearson correlation = 0.87, Supplementary Fig. S4B). Thus, we anticipate that a third dose immunization with stronger immunogenicity (e.g. sequential vaccination with a different type of vaccine) will likely provide adequate protection against the variant virus in non-responders. In sum, we conclude that a third dose of inactivated vaccine should be considered to ensure that most of the vaccinated population acquire sufficient humoral immunity, and trials with sequential vaccination using different types of vaccine, particularly vaccines targeting variants of concern, are encouraged in order to achieve a stronger antibody response for protection against SARS-CoV-2 variants. This study is limited by its relatively small sample size and therefore cannot completely explore the mechanism behind the inter-individual variation of immune response to a third dose among non-responders. Future studies with a larger cohort will likely provide deeper mechanistic insights.

## Supplementary Material

nwac066_Supplemental_Files
